# Analysis of four scales for global severity evaluation in Parkinson’s disease

**DOI:** 10.1038/npjparkd.2016.7

**Published:** 2016-05-05

**Authors:** Pablo Martínez-Martín, Jose Manuel Rojo-Abuin, Mayela Rodríguez-Violante, Marcos Serrano-Dueñas, Nélida Garretto, Juan Carlos Martínez-Castrillo, Víctor Campos Arillo, William Fernández, Pedro Chaná-Cuevas, Tomoko Arakaki, Mario Alvarez, Ivonne Pedroso Ibañez, Carmen Rodríguez-Blázquez, Kallol Ray Chaudhuri, Marcelo Merello

**Affiliations:** 1 National Center of Epidemiology and CIBERNED, Carlos III Institute of Health, Madrid, Spain; 2 Department of Statistics, Centre of Human and Social Sciences, Spanish Council for Scientific Research, Madrid, Spain; 3 Movement Disorders Unit, Instituto Nacional de Neurologia y Neurocirugia, Mexico , Mexico; 4 Movement Disorder and Biostatistics Units, Neurological Service, Carlos Andrade Marín Hospital and Medicine Faculty, Pontifical Catholic University of Ecuador, Quito, Ecuador; 5 Department of Neurology, Hospital Ramos Mejia, Centro Universitario de Neurología de la Universidad de Buenos Aires (UBA), Buenos Aires, Argentina; 6 Movement Disorders Unit, Department of Neurology, Hospital Ramon y Cajal, IRYCIS, Madrid, Spain; 7 Movement Disorders Unit, Department of Neuroscience, Hospital Xanit International, Benalmádena (Málaga), Spain; 8 Movement Disorders Unit, Department of Neurology, Universidad Nacional de Colombia, Bogotá, Colombia; 9 CETRAM, Facultad de Ciencias Médicas, Universidad de Santiago de Chile, Santiago, Chile; 10 Department of Movement Disorders and Neurodegeneration, CIREN, La Habana, Havana, Cuba; 11 National Parkinson Foundation International Centre of Excellence, King’s College Hospital, King’s College, London, UK; 12 Movement Disorders Section, Raul Carrea Institute for Neurological Research (FLENI), Buenos Aires, Argentina

## Abstract

Global evaluations of Parkinson’s disease (PD) severity are available, but their concordance and accuracy have not been previously tested. The present international, cross-sectional study was aimed at determining the agreement level among four global scales for PD (Hoehn and Yahr, HY; Clinical Global Impression of Severity, CGIS; Clinical Impression of Severity Index, CISI-PD; and Patient Global Impression of Severity, PGIS) and identifying which of them better correlates with itemized PD assessments. Assessments included additional scales for evaluation of the movement impairment, disability, affective disorders, and quality of life. Spearman correlation coefficients, weighted and generalized kappa, and Kendall’s concordance coefficient were used. Four hundred thirty three PD patients, 66% in HY stages 2 or 3, mean disease duration 8.8 years, were analyzed. Correlation between the global scales ranged from 0.60 (HY with PGIS) to 0.91 (CGIS with CISI-PD). Kendall’s coefficient of concordance resulted 0.76 (*P*<0.0001). HY and CISI-PD showed the highest association with age, disease duration, and levodopa-equivalent daily dose, and CISI-PD with measures of PD manifestations, disability, and quality of life. PGIS and CISI-PD correlated similarly with anxiety and depression scores. The lowest agreement in classifying patients as mild, moderate, or severe was observed between PGIS and HY or CISI-PD (58%) and the highest between CGIS and CISI-PD (84.3%). The four PD global severity scales agree moderately to strongly among them; clinician-based ratings estimate PD severity, as established by other measures, better than PGIS; and the CISI-PD showed the highest association with measures of impairment, disability, and quality of life.

## Introduction

Patient assessment integrates the data coming from health professional interview and examination, ancillary proofs (biological assays, performance measures, and imaging), self-assessment by patients and, eventually, by proxy evaluations. The combination of findings from these sources supports the diagnosis and allows determining the severity of the disorder with therapeutic and prognostic purposes. Although standardized thresholds for allocation of the severity degree can be available for some results, the assignment of a global severity level is frequently carried out in an intuitive manner based on previous experience and the observed data. To help in this purpose, some ‘global impression’ instruments have been developed and are widely applied.^1–3^

Global evaluations of severity intend providing holistic information on the severity of the disorder which is convenient, for example, for recording and sharing information, classification of patients for resources assignment, and other purposes. The main advantage of this kind of assessment is the approach to an immediate overall appraisal of the health state, whereas their main disadvantage is the lack of detailed information on the health state components.

Parkinson’s disease (PD) is a complex neurodegenerative disorder manifested through typical motor impairment and complications, a diversity of non-motor symptoms, and progressive disability. Observable signs (e.g., tremor or freezing) and performance measures are assessed by a professional rater, but the evaluation of symptoms (e.g., pain, anxiety) and other subjective aspects (e.g., quality of life, satisfaction with care) needs the input of patients. However, it is recognized that perceptions on the patients’ condition frequently differ between patients themselves and their doctors,^4,5^ making difficult sometimes to decide which of these evaluations is more reliable. Determining the severity level of PD is a challenge not only by these discrepancies, but also because the expression of the disorder greatly varies from case to case in components and their relative importance.

In a previous study,^6^ we collected information from four global scales to determine cutoff points for assignment of primary severity levels to the scores of the Movement Disorder Society sponsored version of the Unified Parkinson’s Disease Rating Scale (MDS-UPDRS). These four scales were the Hoehn and Yahr staging (HY),^7^ the Clinical Global Impression-Severity (CGIS),^1^ the Patient Global Impression-Severity (PGIS),^2,8^ and the Clinical Impression of Severity Index for Parkinson’s disease (CISI-PD).^9,10^ Two of these instruments are generic (CGIS and PGIS) and the other two are specific for PD (HY and CISI-PD). On the other hand, three are single-item scales (HY, CGIS, and PGIS) and one is multi-item scale (CISI-PD).

The objective of the present study were: (1) to determine the degree of agreement among these four global severity scales and (2) to identify which of them is more closely associated to the other variables in the study reflecting severity of PD-related aspects.

Our working hypotheses were: (1) the four scales for global assessment of PD are dissimilar in source of information and content; therefore, the agreement between scales will be only partial and different between rater-based and patient-based evaluations (objective 1), and (2) if the global scales differ among them, one of them could be more strongly associated with other markers of disease severity and, consequently, could be deemed the most accurate to estimate the global severity of PD (objective 2). No prospects on the results with any particular scale were advanced for this second hypothesis.

## Results

From 452 included PD patients, 433 (95.8%), 54.5% males, were considered for analysis once 19 cases with missing values or errors (two responses for one item or values out of range) were dropped out. The distribution by HY was: stage 1, 13.9%; stage 2, 36.7%; stage 3, 30.2%; stage 4, 15.7%; and stage 5, 3.5%. The demographic characteristics and PD historical data are summarized in the Table 1, whereas the Table 2 shows the distribution of the sample as per the severity levels determined for each scale. The maximum disagreement was found for PGIS with HY and CISI-PD, which were coincident in severity level only in 58.89% of patients (sensitivity analysis: confidence interval=4.63, for a 95% confidence level).

Table 3 displays the correlation between the global scales, which ranges from 0.60 (HY with PGIS) to 0.91 (CGIS with CISI-PD). As a whole, highest values were obtained with the CISI-PD for all correlations. CGIS and CISI-PD were highly correlated (*r*_S_=0.61–0.78) across all HY stages except stage 1 (*r*_S_=0.40), whereas PGIS showed a moderate correlation (*r*_S_=0.30–0.56, except for HY stage 5, *r*_S_=−0.02) with the CISI-PD and a weak correlation with the CGIS (except for HY stage 4, *r*_S_=0.41).

Concerning the concordance of the four global scales to classify patients as mild, moderate or severe, the lowest percentage of agreement was observed between PGIS with HY and CISI-PD (58%) and the highest between CGIS and CISI-PD (84.3%). Weighted kappa followed a similar behavior (Table 4). Kendall’s coefficient of concordance for the severity levels of the four scales resulted 0.67 (*P*<0.0001) and the generalized kappa statistic was 0.52 (95% confidence interval (CI)=0.64–0.73).

The correlation coefficients between the four global scales and the other variables in the study are displayed in the Table 3. As a whole, HY and CISI-PD had the highest association with time-related variables (age and duration) and levodopa-equivalent daily dose. CISI-PD reached the highest coefficients with measures of PD manifestations severity (MDS-UPDRS), disability (SES and BI), and quality of life (EQ-5D-3L, EQ-VAS, and PDQ-8). PGIS and CISI-PD correlated similarly with HADS-A and HADS-D, at a higher level than HY and CGIS.

## Discussion

This study was focused in analyzing the performance of four simultaneously applied global assessments based on the clinician (HY, CGIS and CISI-PD) and patient (PGIS). Two of these assessments are specific PD evaluations (HY and CISI-PD) and the other two are generic (CGIS and PGIS). They have different ranges of scores, running from 0 to 5 for HY and PGIS to 0 to 24 (CISI-PD). Nonetheless, the four scales can be converted to a classification in three severity levels immediately interpretable: mild; moderate; and severe. This shared ordinal scaling allowed analyses to determine the degree of agreement among scales, which is influenced not only by the differences in structure and scoring procedure, but also by the origin of the information (patient versus clinician). In this sense, it could be expected that rater-based global scales showed higher concordance with the rater-based evaluations, whereas the patient global impression would be more concordant with patient-reported outcomes.

Our results concerning the first hypothesis demonstrate that:

1. The four scales are closely correlated between them, although with variable strength: the strongest correlation was observed between CGIS and CISI-PD, whereas the weakest was observed between clinician-based scales and patient self-evaluation (Table 3). Previous studies have reported a substantial agreement between functional evaluations performed by patients and clinicians,^11,12^ although patients tend to overestimate their functional disability in comparison with doctors’ evaluations.^5^ These relationships can be complex and depend on circumstances such as comorbidities or the availability of a caregiver.^5,12,13^
2. The concordance among severity levels derived from the tested scales showed percentages of agreement from 58% (PGIS with HY and with CISI-PD) to 84.3% (CGIS with CISI-PD) and kappa values from 0.57 (moderate, PGIS with CISI-PD) to 0.86 (almost perfect, CGIS with CISI-PD)^14^ (Table 4), findings replicating those of the correlations commented above. Both, generalized kappa and Kendall’s concordance coefficient showed a moderate to strong agreement amongst the classification in severity levels from the four scales, indicating they are not equivalent at all.

In summary, (1) the four scales show a moderate to strong association among their scores and concordance on the severity levels classification derived from them (Tables 3 and 4); (2) the analyzed global scales are not equivalent, and (3) the patient-reported evaluation correlates at a lower degree with the rater-based scales.

Regarding the second hypothesis and once demonstrated that the tested global scales are not equivalent, is there any of them more closely associated, as a whole, to the diverse measures assessing different aspects of PD? From a logical point of view, the scale achieving the closest association with all the other detailed measures reflecting PD severity and impact (motor impairment and complications, non-motor symptoms, disability, quality of life), both from clinical evaluation and patient self-assessment could be deemed the most appropriate for the overall evaluation of PD severity. As shown in the Table 3, the CISI-PD was, as a whole, the scale most closely correlated with any other variable measuring severity of PD manifestations, functional disability, and impact on quality of life. The latter is a remarkable finding, as it would be expected that patient’s self-evaluation would be more closely correlated with the patient-reported outcomes than rater-based assessments. In fact, only the anxiety evaluation reached higher correlation values with the PGIS than with clinician-based assessments (Table 3).

When other global scale reached a higher correlation value with other variables than the CISI-PD (HY for age, PD duration, and levodopa-equivalent daily dose; PGIS for anxiety) the difference was 0.01–0.03. However, CISI-PD coefficients reached values up to 0.11 and 0.24 higher than the other rater-based global scales and PGIS, respectively (Table 3). Although these differences are not huge, the constant closer association results suggest that the CISI-PD would be the most appropriate instrument for global estimation of PD severity.

PD is a complex disease with motor and non-motor symptoms and complications. Global evaluations can provide a comprehensive appraisal of the health state in a rapid manner, but HY is based only in motor signs and functioning^15^ and CGIS and PGIS are overall valuations, too broad, unspecific, and with a marked subjective component difficult to control.^16^ The CISI-PD offers a more detailed estimation of PD-related global health from four outstanding aspects of the disorder that can be individually monitored with this pragmatic instrument. It is designed to be scored after the interview and examination have been carried out, in order to capture information enough and decrease the judgment based on the mere patient’s appearance.^9,10^ In addition, CISI-PD has a higher precision (range, 0 to 24 points) than the other scales (1 to 7 with the CGIS or 1 to 5 with the HY and PGIS). In spite of the CISI-PD is composed of four items, it may be considered a global evaluation because gathers the overall clinical impression on the corresponding domains and takes seconds to be completed. Previous studies found that CISI-PD explains 92% of the CGIS variance and as a whole, correlated at higher level than other global measures with a diversity of scales assessing a diversity of PD-related manifestations and consequences.^9,10,17,18^

Limitations of the study are: (1) most of patients in the sample were in HY stages 2 and 3, with stages 1, 4, and 5 relatively little represented, a shortage usual in clinical samples with consecutive patients from specialized settings (departments of Neurology, specialized units); nonetheless, there were 83 patients in the HY-based level with the lowest representation (‘severe’); (2) the selection of patients, as patients with dementia were excluded; the need of reliable patients’ introspection justifies this limitation, although cognitive state is of marginal importance for HY staging; (3) another potential limitation is related to variability depending on multiple sites and raters, although the group has experience in previous collaborations. On the other hand, the studied scales are used in similar conditions as they were applied for this study and, therefore, represent appropriately their use in daily practice and applied research.

The characteristics of the study, with different countries and researchers contributing, the size adjusted to acceptable levels of certainty, and a sample characteristic of the specialized background in which the field work was carried out (and where its outcomes will be applied) allow foreseeing close findings in future studies on the topic with the instruments applied here.

Conclusions are: (1) the four scales applied to estimate the global health state of PD patients are moderately to strongly related among them and agree in their estimations moderately to almost perfectly; (2) as expected, the clinician-based assessments correlate closely between them and higher than with patient self-evaluation; and (3) the CISI-PD shows, as a whole, the tightest association with other measures of PD focused on motor and non-motor impairment and complications, disability and, even, patient-reported health-related quality of life assessments.

## Materials and methods

### Design

Multicenter, international, observational, cross-sectional study.^6^ Nine centers from seven countries (Argentina, Chile, Colombia, Cuba, Ecuador, Mexico, and Spain) participated in the sample recruitment. Two centers, in Spain and United Kingdom, carried out the organization of the study and statistical analysis. Data were collected from February to September, 2013

### Patients

Consecutive patients were included if they were diagnosed with PD by a neurologist competent in movement disorders, according to international criteria.^19^ Exclusion criteria were: (1) parkinsonian syndrome different to PD; (2) presence of any disabling condition impeding or interfering with the evaluation of PD.

Patients with more than mild cognitive deterioration were excluded from the analysis. The operational criterion to this purpose was: scoring ⩾3 in the item 1 of the MDS-UPDRS Part I and ⩾4 in the item Cognition of the CISI-PD. Patients with problems to answer written questionnaires (e.g., due to visual difficulties or action tremor) were assisted by a trained person.

General population in the participant countries is around 303.5 million, with around 1 million of patients suffering PD.^20^ Sample size for this study was calculated as a survey representative of PD population with a confidence level 95%, a s.d. 0.5, and a confidence interval=5. To this purpose, 384 fully analyzable patients must be included. An additional 10% was agreed in prevention of the missing data or mistakes. Therefore, a minimal total sample 422 was proposed.

### Ethics issues

The study was approved by the local Ethics Committee or Institutional Review Board of each participant site. Patients gave their signed consent to participate in the study.

### Assessments

Demographic and PD historical information were collected through interview and clinical records and the following assessments were used:

*MDS-UPDRS Spanish version*:^21^ this is a multidimensional measure for assessment of Non-Motor Experiences of Daily Living (Part I; 13 items: six rater-based and seven self-assessed by patient and/or by proxy), Motor Experiences of Daily Living (Part II; 13 patient-based items); Motor examination (Part III; 18 rater-based items with 33 scores); and Motor complications (Part IV; 6 items, rater-based). Each item scores from 0 (normal) to 4 (severe) and total scores for each of the four domains are obtained from the sum of the corresponding item scores.

*HY*:^7^ the original version of this classification (1, Unilateral involvement only usually with minimal or no functional disability; to 5, Confinement to bed or wheelchair unless aided) was used, as recommended.^15^

*Global impression of severity*: the clinician-based (CGIS), with 7 response options^1^ and a 6-option patient-based (PGIS),^6^ with the option ‘severe’ collapsing the ‘markedly ill’ and ‘severely ill’ options, were applied in the study.

*CISI-PD*:^9,10^ a clinical estimate of PD severity based on the impression of the clinician about the severity of four outstanding PD aspects: motor signs; disability; motor complications; and cognitive status. Each item-domain scores from 0 (normal) to 6 (very severe) and a total score ranging from 0 to 24 can be calculated.

*Schwab and England Scale (SES)*:^22^ a rater-based measure of functional independence ranked according to eleven options (from 100%, completely independent, to 0%, completely dependent and bedridden). Scores are obtained by interview and observation.

*Barthel Index (BI)*:^23^ a generic, widely used assessment of functional independence for activities of daily living. It is composed of 10 items and the total score ranges from 0 to 100.

*Hospital Anxiety and Depression Scale (HADS)*:^24^ a self-rated scale for global assessment of mood disorder. It consists of 7 items for evaluation of anxiety and seven for depression. Each item scores from 0 (no problem) to 3 (severe problem). Scores of individual items can be summed to calculate separate scores for anxiety (HADS-A) and depression (HADS-D). The HADS is adequate for use in PD patients.^25^

*EQ-5D-3L*:^26^ a measure of health status providing a descriptive profile and a single index value (from 0 to 1) representing the global quality of life (preference) for clinical and economic evaluation. It has 5 items, each with 3 possible response levels. Higher scores represent worse perceived health. In addition, a visual analog scale (EQ-VAS) from 0 (the worst) to 100 (the best) is used to assess the global ‘health status today’. The EQ-5D is ‘recommended’ for use in PD.^27^

*Parkinson’s Disease Questionnaire—8 items (PDQ-8)*:^28^ it includes 8 items, each representing a dimension of the mother health-related quality of life questionnaire PDQ-39. A summary index is obtained by summing the 8 items and standardizing on a scale of 0–100. Higher scores reflect worse HRQoL. It has been ‘recommended’ for use in PD.^27^

### Data analysis

Central tendency and dispersion measures (mean, s.d., 95% CI, range), as well as proportions, were calculated for description of the variables in the study. Levodopa-equivalent daily dose was calculated as per Tomlinson *et al.* ^29^

The paired correlation between the four global severity scales was determined by means of the Spearman rank correlation coefficient, as they did not meet assumptions for parametric tests.

Scores of the global severity scales were transformed to three severity levels (mild, moderate, and severe) according to previous studies^10,15^ or the response options wording itself, as follows: (1) HY: stages 1 and 2, mild; stage 3, moderate; and stages 4 and 5, severe;^15^ (2) CISI-PD: 1–7, mild; 8–14, moderate; ⩾15, severe;^10^ (3) CGIS: 2–3, mild; 4, moderate; 5–7, severe; and (4) PGIS: 1–2, mild; 3, moderate; 4–5, severe. The agreement among these levels for each pair of scales was determined by percentage and weighted kappa (with quadratic weights), and for all of them simultaneously by Kendall's coefficient of concordance and generalized kappa.^30^

The correlation with other variables in the study (clinical ratings and patient-reported outcomes) was explored by means of the Spearman rank correlation coefficient. Correlation coefficient values <0.30 were considered weak correlation and ⩾0.60, strong correlation.^31^

## Figures and Tables

**Table 1:** Descriptive data of the sample demography and assessment

	*Mean*	*s.d.*	*95% CI*	*Range*
Age at study	65.1	10.7	64.1–66.1	22–91
Age at Parkinson’s disease onset	56.4	11.2	55.4–57.5	17–80
Parkinson’s disease duration	8.8	6.4	8.2–9.4	0–40
Years since diagnosis	7.9	6.1	7.4–8.5	0–37
Duration of treatment	7.7	6.1	7.1–8.3	0–37
Movement Disorder Society—UPDRS 1	11.1	7.0	10.6–2.0	0–34
Movement Disorder Society—UPDRS 2	15.7	11.4	14.6–16.7	0–49
Movement Disorder Society—UPDRS 3	35.7	21.1	33.7–37.7	0–97
Movement Disorder Society—UPDRS 4	4.8	5.0	4.3–5.2	0–18
Schwab and England scale	73.8	21.0	71.8–75.8	10–100
Barthel index	84.9	22.6	82.7–87.0	0–100
Hospital Anxiety Depression Scale—anxiety	7.4	3.9	7.1–7.8	0–20
Hospital Anxiety Depression Scale—depression	6.5	4.3	6.1–6.9	0–20
EQ-5D-3L	0.62	0.33	0.59–0.66	−0.65–1
EQ Visual analog scale	63.6	23.2	61.4–65.8	0–100
Parkinson’s disease questionnaire—8 items	29.7	22.0	27.6–31.7	0–100
CISI-PD	8.6	5.1	8.1–9.1	1–21
Levodopa equivalent daily dose	774.2	488.4	728.1–820.3	0–4000

Abbreviations: CI, confidence interval; CISI-PD, Clinical Impression of Severity Index for Parkinson’s disease; EQ, EuroQoL questionnaire; EQ-5D-3L, EuroQoL questionnaire, 5 dimensions, 3 levels; UPDRS, Unified Parkinson’s Disease Rating Scale.

**Table 2:** Distribution of patients by severity levels according the global scales

	*Hoehn and Yahr staging*	*Clinical impression of severity index* a	*Clinical global impression of severity*	*Patient global impression of severity*
Mild	50.6%	44.8%	47.6%	40.2%
Moderate	30.3%	35.1%	37.0%	38.6%
Severe	19.2%	20.1%	15.5%	21.3%

a Clinical Impression of Severity Index for Parkinson’s Disease.

**Table 3:** Correlation between the global assessments and with the other variables in the study

*Between scales correlation*	*Hoehn and Yahr staging*	*Clinical global impression—Severity*	*Clinical impression of severity index* a	*Patient global impression of severity*
Clinical global impression—Severity	0.80	—	—	—
Clinical Impression Severity Index^a^	0.83	0.91	—	—
Patient global impression severity	0.60	0.61	0.66	—
				
*Correlations with other variables*				
Age at study	0.28	0.22	0.25	0.18
Age at PD onset	−0.05	−0.08	−0.08	−0.03
PD duration	0.61	0.54	0.61	0.37
Years since diagnosis	0.59	0.53	0.59	0.35
Duration of treatment	0.58	0.52	0.59	0.35
MDS-UPDRS 1	0.58	0.57	0.66	0.55
MDS-UPDRS 2	0.76	0.80	0.85	0.65
MDS-UPDRS 3	0.69	0.70	0.76	0.61
MDS-UPDRS 4	0.68	0.69	0.80	0.56
Schwab and England scale	−0.81	−0.81	−0.85	−0.66
Barthel index	−0.67	−0.68	−0.72	−0.54
Levodopa equivalent daily dose	0.44	0.35	0.43	0.32
HADS—Anxiety	0.41	0.43	0.47	0.49
HADS—Depression	0.46	0.50	0.55	0.54
EQ-5D–3L	−0.62	−0.67	−0.72	−0.62
EQ Visual analog scale	−0.55	−0.58	−0.62	−0.60
PDQ-8	0.66	0.70	0.74	0.67

Abbreviations: EQ, EuroQoL questionnaire; 5D–3L, 5 dimensions, 3 levels; HADS, Hospital Anxiety and Depression Scale; MDS-UPDRS, Movement Disorder Society-Unified Parkinson’s Disease Rating Scale; PD, Parkinson’s disease; PDQ-8, Parkinson’s Disease Questionnaire—8 items.

a Clinical Impression of Severity Index for Parkinson’s disease.

**Table 4:** Agreement between severity levels of the four scales

	*Hoehn and Yahr staging*	*Clinical global impression of severity* a	*Clinical impression of severity index*
Clinical Global Impression of Severity	78.5% (0.81; 0.79–0.82)	—	—
Clinical Impression of Severity Index^a^	79.0% (0.80; 0.79–0.82)	84.3% (0.86; 0.84–0.87)	—
Patient Global Impression of Severity	58.0% (0.58; 0.53–0.61)	61.0% (0.63; 0.60–0.67)	58.0% (0.57; 0.54–0.61)

%: percentage of agreement. Between brackets, weigthed kappa and confidence intervals 95%.

a Clinical Impression of Severity Index for Parkinson’s disease.
